# Evolution of community pharmacy services in the European Union and beyond: a cross-country survey of 33 national pharmacy organisations

**DOI:** 10.1007/s11096-026-02137-9

**Published:** 2026-04-15

**Authors:** Jorge P. B. Batista, Anita Elaine Weidmann, Erika Mallarini, Martin C. Henman, Ilaria Passarani

**Affiliations:** 1 Pharmaceutical Group of the European Union (PGEU), Brussels, Belgium; 2 Universität Innsbruck, Innsbruck, Austria; 3 SDA Bocconi School of Management, Milan, Italy; 4 Trinity College Dublin, Dublin, Ireland; 5 Universidade Nova de Lisboa, Instituto de Higiene e Medicina Tropical, Lisbon, Portugal

**Keywords:** Community pharmacy, Community pharmacy services, Europe, Health policy, Reimbursement, Service implementation

## Abstract

**Introduction:**

Community pharmacy practice in Europe has evolved, expanding beyond dispensing roles to include preventive, counselling, and clinical services. However, evidence on the implementation, reimbursement, and regulation of these services across the European Union (EU) remains limited, especially after the COVID-19 pandemic. Understanding these developments is crucial to inform workforce planning, funding frameworks, and the integration of pharmacists into primary care. With limited healthcare resources, investing in pharmacy-based public health interventions leverages pharmacies’ accessibility, geographic distribution, trusted patient relationships, and skilled workforce.

**Aim:**

This study aimed to update the mapping of community pharmacy services (CPS) across Europe, comparing 2025 data with pre-pandemic (2020) findings, and to examine trends in service provision, reimbursement, and regulatory barriers from a health policy perspective.

**Method:**

A cross-sectional survey was conducted among national pharmacy associations in all 27 EU Member States and six neighbouring countries (n = 33), to contextualise EU findings within the wider European pharmacy practice landscape. A previously validated instrument was used to collect data on 47 CPS, including implementation level and reimbursement status. Responses were validated through national review, and data were analysed descriptively and compared with 2020 results.

**Results:**

Across the 33 countries, a median of 26 services per country was reported (range: 9–43). Using a consistent data collection instrument and methodology, 77% (n = 36) of mapped CPS were available in a greater number of countries in 2025 than in 2020. Notable growth was observed in vaccination (10–19 countries), medication reconciliation (7–15), and first-time dispensing interventions (11–16). Public reimbursement increased for 49% (n = 23) of services between 2020 and 2025, with the largest increases in vaccination (+ 5 countries), screening (+ 4 countries), and dose administration aids (+ 3 countries). Despite this progress, variation between countries persists, alongside regulatory and operational barriers (remuneration, workforce capacity and interprofessional integration).

**Conclusion:**

Since 2020, CPS have expanded across Europe, with wider availability and, in some cases, progression from pilots or individual provision to national implementation. Wider and more sustainable implementation appears to depend on supportive regulatory frameworks and public remuneration.

**Supplementary Information:**

The online version contains supplementary material available at 10.1007/s11096-026-02137-9.

## Impact statements

- The expansion of community pharmacy services in Europe highlights opportunities for pharmacists to assume wider clinical roles within primary care, particularly in vaccination, medicines optimisation, and prevention services.
- Policymakers and healthcare managers should align regulatory frameworks and remuneration models to match the expanding scope of pharmacy practice to ensure sustainable implementation.
- Cross-country heterogeneity in service availability and funding indicates a need for greater policy coordination and exchange of best practices across Europe.
- International health system assessments (e.g., OECD reports) should better reflect the contribution of community pharmacy services through dedicated indicators, supporting cross-country benchmarking and enabling evidence-informed policy development.
- Strengthening workforce capacity and interprofessional collaboration will be essential to translate service availability into consistent patient benefit.

## Introduction

Community pharmacy practice has expanded considerably over recent decades, adapting to meet evolving societal needs and contributing significantly to public health development [1]. The role of community pharmacists has extended far beyond traditional medicines dispensing, to include a wide range of clinical, preventive, and counselling services that support patient care [2, 3]. Although pharmacy practice is broadly regulated in the European Union (EU), pharmacists’ involvement in patient care is not yet fully reflected in existing legislation, nor recognised by healthcare services [4]. Expanding the pharmacists’ scope of practice has occurred in a context of gaps in primary care coverage, budgetary constraints, efficiency pressures, and emerging public health challenges, requiring more optimal use of healthcare resources, including the management of an increasing number of innovative medicines [5–11]. Against this backdrop of system pressures and expanding professional roles, understanding how community pharmacy services are implemented, regulated and funded in the European region has become progressively relevant from a health policy perspective.

Population ageing, evolving patients’ expectations, chronic disease burden, and constrained health budgets are increasing pressure on European health systems, thus reinforcing the need for a stronger primary care and efficient use of health resources [3, 12, 13]. In response, community pharmacies are shifting their focus from dispensing medicines and managing diseases, to further patient care and service quality [14, 15]. This encompasses regulatory changes, organisational and cultural adaptations [16]. As national legislation has evolved, pharmacists have taken on differentiated roles responding to community needs, a trend further accelerated during and after the COVID-19 pandemic through vaccination, prescription renewals, home delivery of medicines and, in some countries, medicine shortages mitigation measures (such as generic/therapeutic substitution, compounding, dosage adaptation) [1, 17–26]. However, evidence from practice suggests that services are not uniformly accessible across Europe [27].

For the purpose of this study, community pharmacy services were defined as pharmacist-led public health interventions in community pharmacy settings spanning health promotion, medication management, and disease prevention. In some countries, selected services may be also performed by trained pharmacy technicians, such as medicines supply services; however, this study focused only on services delivered by pharmacists [28, 29]. Pharmacist-led services such as medication management, clinical, and prevention services are intended to enhance the quality of care, improve health outcomes and lower costs [30–32]. In particular, over the last decade there has been an increase in services related to vaccination, point-of-care testing, smoking cessation, chronic disease management and health screening and assessment [18]. For the purposes of this study, services were grouped into six categories: dispensing services, health promotion services, screening and referral services, disease management services, individual case management services, and services based on Health Technology Assessment (HTA) and digital services.

International stakeholders, the World Health Organization (WHO) Europe, Organisation for Economic Co-operation and Development (OECD) and the European Commission have all acknowledged that community pharmacists are the most accessible healthcare professionals to the public and that they could provide more services [4, 16, 26, 33]. The OECD’s pre‑COVID assessment highlighted struggling health systems and identified stronger primary health care as key to improving access and quality, and further identified broader roles for community pharmacists in renewing and modifying prescriptions and health promotion, and measures to support team‑based care and collaboration [34]. These pre-pandemic conclusions remain relevant in the post-pandemic context, where primary care strengthening and resilience have become explicit policy priorities in Europe [26, 33]. Within healthcare systems’ limited resources, it is crucial to invest in public health interventions that maximise accessibility and equity by taking advantage of pharmacies’ geographical distribution, regular patient interaction with increased trust, extended opening hours with walk-in access, and highly skilled pharmacist workforce [35, 36]. Recent research has explored the economic impact of community pharmacy services, reviewing and proposing remuneration models [28, 37, 38]. Economic evaluations systematically indicate that community pharmacy services can be cost-effective and may reduce pressure on primary care, with positive impacts on health systems budgets [39–42]. Comparative and post-pandemic evidence on the implementation, reimbursement and regulatory frameworks for community pharmacy services in all EU countries remains limited. The Institute for Evidence Based Health (ISBE) report on pharmacy services (2020) provided important baseline data, but did not include all 27 EU Member States [18]. The present study assesses post-pandemic developments and offers insights on reimbursement trends by comparison of data collected across all of Europe using the same tool.

### Aim

The primary aim of this empirical, survey-based study was to map the implementation of community pharmacy services across European countries in 2025, building on comparable 2020 data. The secondary aim was to explore patterns of reimbursement and policy-level regulatory barriers associated with service provision and implementation.

## Method

### Study design

A quantitative repeated cross-sectional survey was conducted in 2025, followed by a single round of data validation. This study included a longitudinal comparative component through a structured comparison with data previously collected using the same instrument and methodology in 2020.

### Population and sample

The inclusion criteria of participants for this study comprised being members (national pharmacy associations and professional bodies of community pharmacists) of the Pharmaceutical Group of the European Union (PGEU) or being a Member State of the EU [43]. All 27 EU Member States and the following countries were invited to participate in the study: Kosovo, North Macedonia, Norway, Serbia, Switzerland, Türkiye, United Kingdom. Seven neighbouring Non-EU countries were invited to participate (six provided data and were included in the final dataset). These countries were included to contextualise EU findings within the broader European pharmacy practice perspective.

### Data collection

A previously validated and published questionnaire was used, covering six main topics: dispensing services, health promotion services, screening and referral services, disease management services, individual case management services, and services based on Health Technology Assessment (HTA) [18]. This instrument was originally developed and validated in the 2020 ISBE study through expert consultation and pilot testing [18]. For the 2025 survey, the same questions, service definitions, response options, categorisation framework, respondent network and validation process were retained. Only limited additions were made to capture newly emerging services since 2020, particularly post-pandemic and digital/HTA-related services. Digital services were grouped with HTA-related services, as both involve structured data collection at community pharmacy level and to allow for comparability with the 2020 categorisation. These additions expanded coverage of emerging services but did not alter the structure used for longitudinal comparison of services already mapped in 2020. The questionnaire was distributed electronically (June–December 2024), via the PGEU internal database (membership list). In countries where more than one national organisation was eligible to participate, representatives jointly prepared a single, harmonized, consolidated national submission. Any discrepancies were resolved at national level prior to submission, resulting in one response per country. Two reminders were issued (8 weeks apart), and no incentives were provided to complete the questionnaire. Respondents could consult their 2020 national responses to ensure continuity, minimize reporting inconsistencies, and ensure response standardization. Reimbursement information was recorded as the presence of government (health insurance/payer) funding for a given service.

### Data extraction and validation

Responses were coded in Microsoft Excel (v 2024). Data extraction was performed by one researcher (JB) in January 2025 and independently verified by a PGEU staff member (LA). A preliminary report was produced and circulated in February 2025 for internal review (IP) and then shared with national respondents in March 2025 for data validation and updating.

### Data analysis

Descriptive statistics (frequencies and distributions) were used to summarise service implementation and reimbursement across countries. In line with the study’s objectives of mapping service implementation and policy characteristics across European countries, descriptive analysis was considered appropriate in order to provide a comparative overview, rather than inferential estimation. Results are presented narratively and in table format. Longitudinal comparison analysis was performed against previously published grey literature that used the same questionnaire, data collection strategy and methodology in 2020 [18]. In order to address variation in implementation intensity, community pharmacy services were categorized according to their level of provision (provided in most pharmacies under contract, agreement, regulation; provided individually by some pharmacies; provided in some pharmacies as a pilot; provided in some pharmacies as a pilot and individually by some pharmacies). Longitudinal comparison with 2020 data therefore reflects both changes in geographical availability of services, as well as shifts in implementation intensity.

### Ethics approval

This study did not require formal ethical approval. It involved aggregated, non-identifiable data provided by national pharmacy organisations and publicly available information. No personal, patient, or sensitive data were collected or processed.

## Results

Authors received replies to the community pharmacy services survey from 33 countries: all 27 EU Members (Austria, Belgium, Bulgaria, Croatia, Czechia, Cyprus, Denmark, Estonia, Finland, France, Germany, Greece, Hungary, Ireland, Italy, Latvia, Lithuania, Luxembourg, Malta, Netherlands, Poland, Portugal, Romania, Slovakia, Slovenia, Spain, and Sweden) + North Macedonia, Norway, Serbia, Switzerland, Türkiye and United Kingdom. Kosovo was invited to participate but did not submit a completed response and was therefore not included in the final dataset.

### Availability of community pharmacy services

**Fig. 1:**
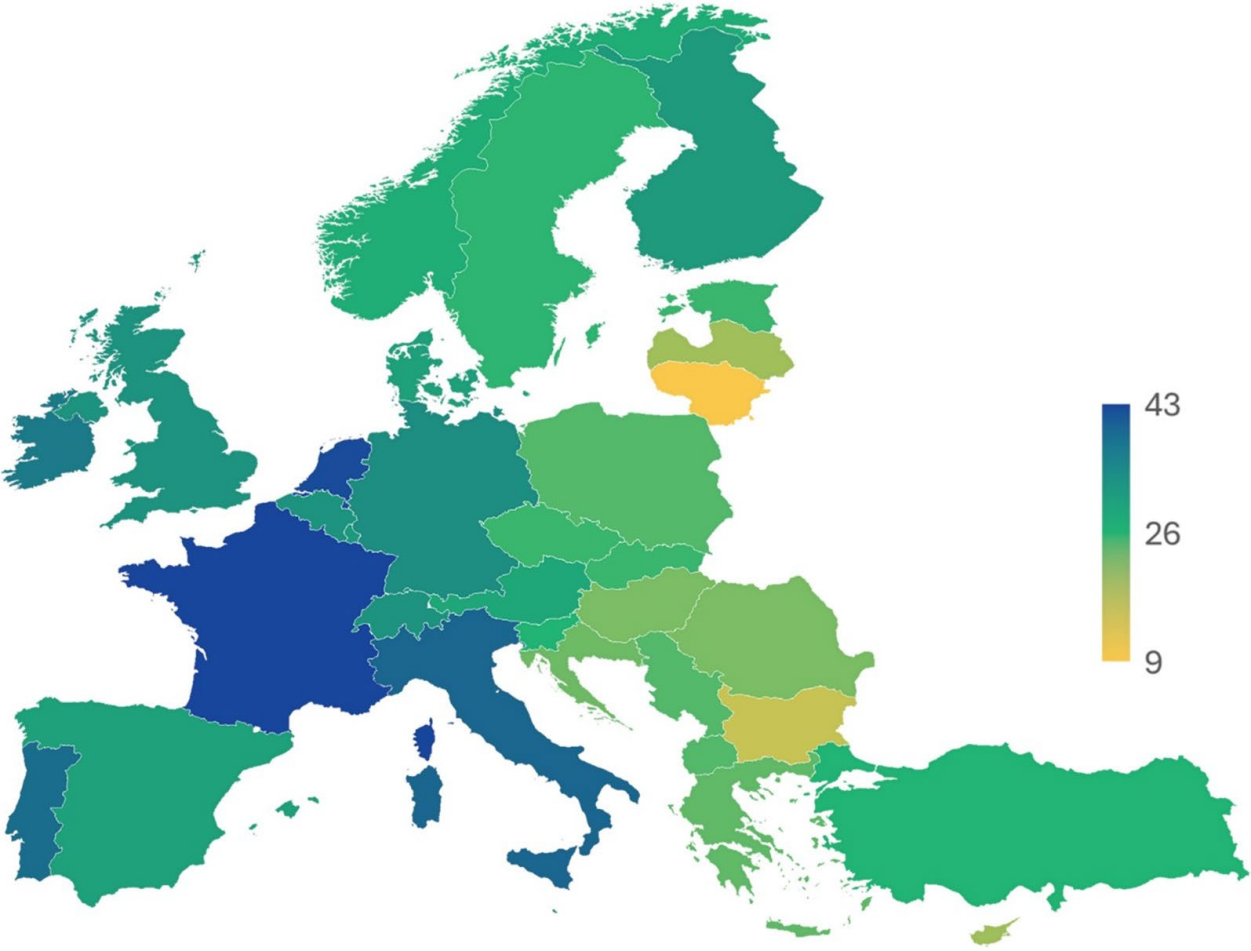
Number of community pharmacy services available in each European country

**Fig. 2:**
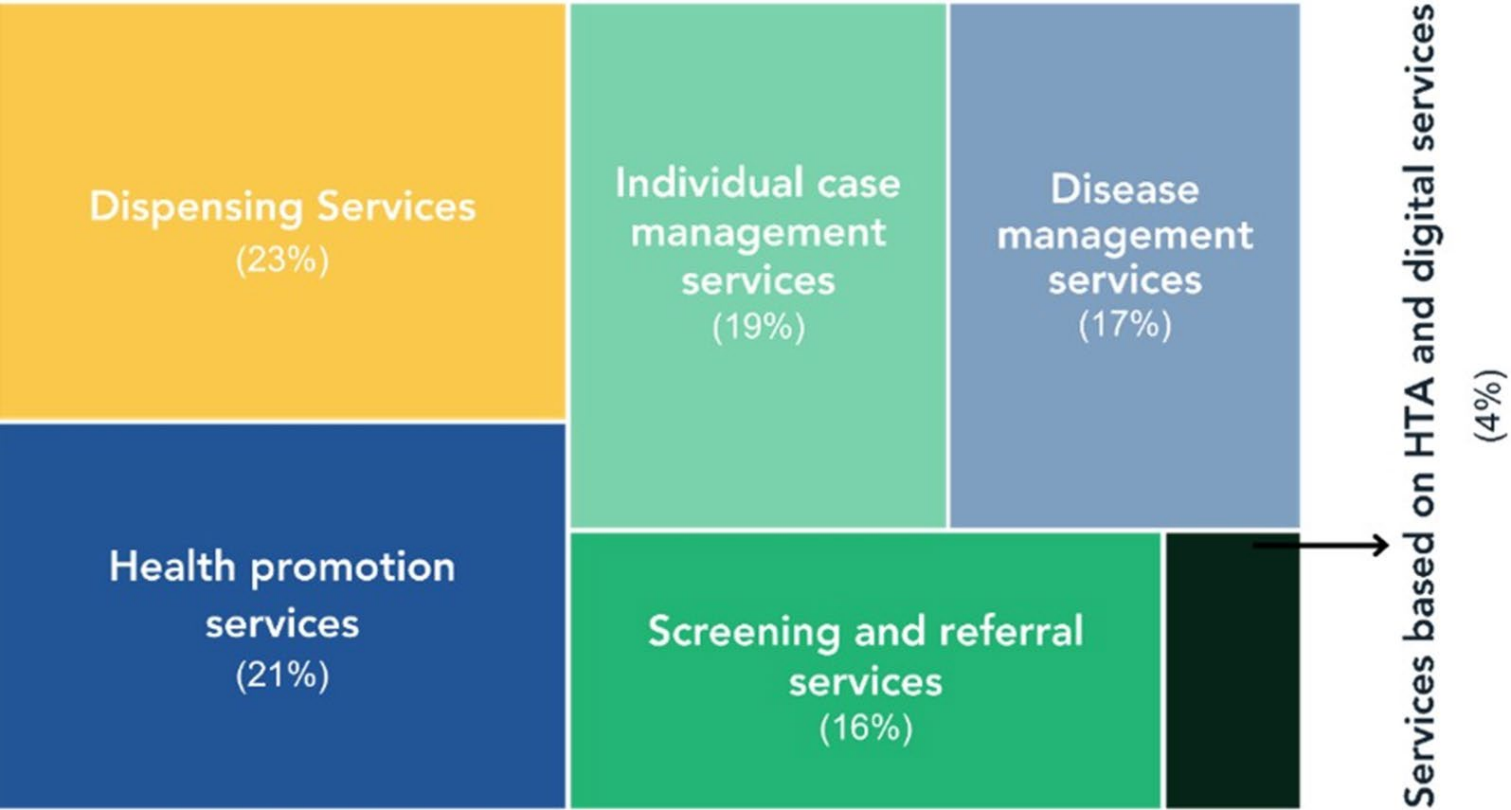
Proportion of each category of community pharmacy services available in Europe

A total of 47 different community pharmacy services were identified across all 33 countries. These were mapped and divided into the following categories: Dispensing related services (n = 8), Health promotion services (n = 7), Screening and referral services (n = 8), Disease management services (n = 8), Individual case management services (n = 12), Services based on HTA and digital services (n = 4). Additionally, a total of 27 country-specific services were coded as “others”. Table 1 details an overview of Community Pharmacy services in Europe in all surveyed countries. Table 2 provides further information on the comparison of the number of existing community pharmacy services in 2020 versus 2025 [18]. Figure 1 provides a synthesis of the frequency of community pharmacy services in Europe through a map, and Fig. 2 details the proportion of each category of community pharmacy services available in Europe.

**Table 1:**
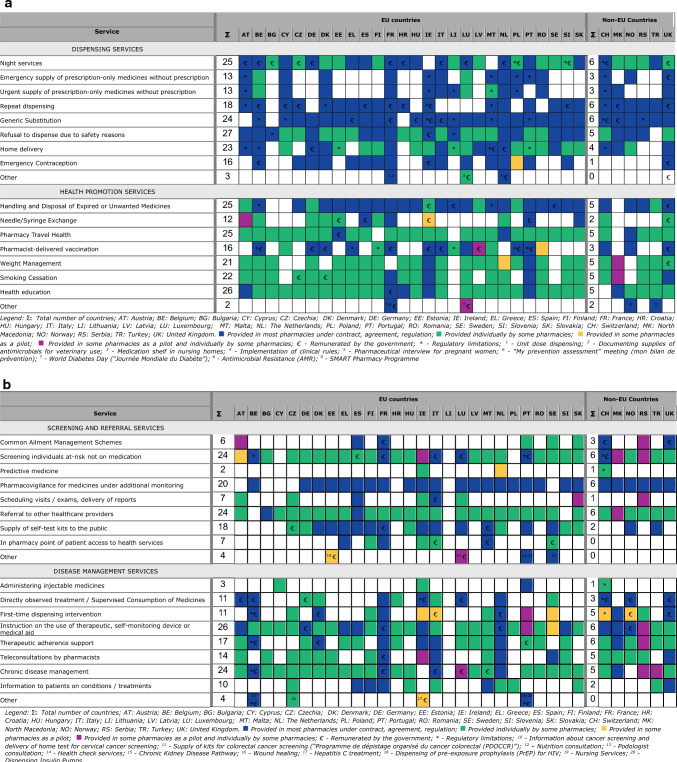

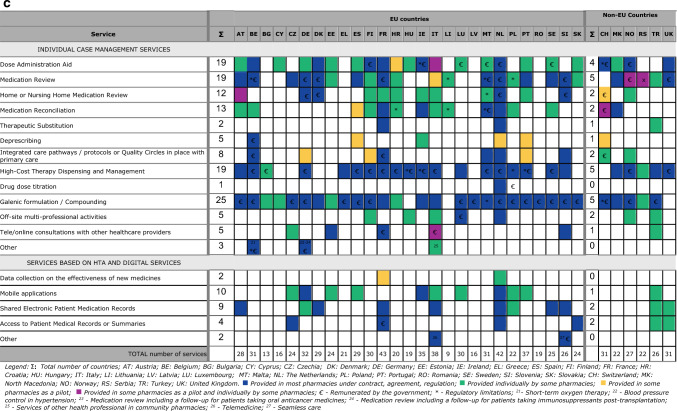
Overview of community pharmacy services in Europe

**Table 2:**
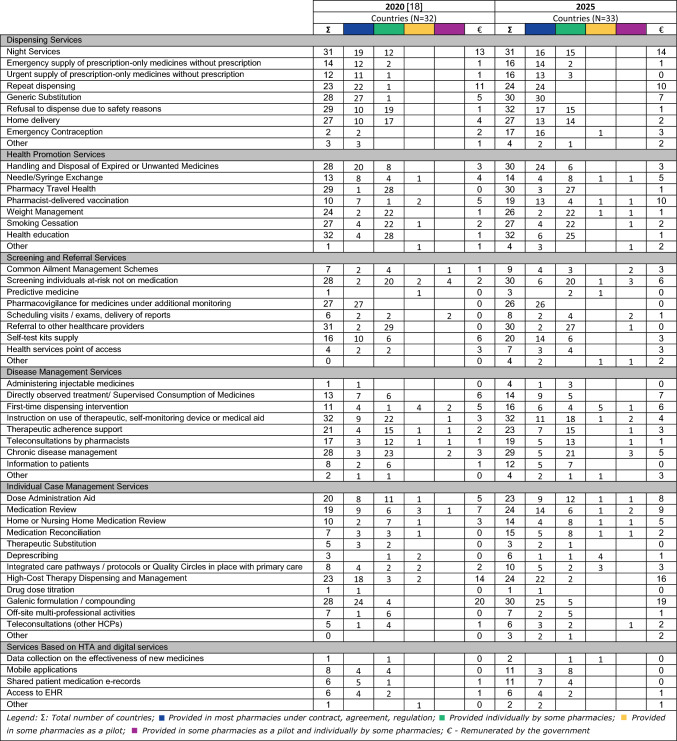
Comparison between data from 2020 to 2025 (number of countries implementing/remunerating each service)

Overall, community pharmacy services availability varied considerably across European countries, with a median of 26 services per country (range 9–43). Disease-management services reveal the greatest relative increase since 2020, whilst dispensing services, which were already largely implemented, remained stable. A small number of services demonstrated stable (or even declining) availability, reflecting national regulatory or organisational differences.

Using the same questionnaire and methodology applied in 2020, comparison of the 2025 data with the previous dataset shows a clear expansion of community pharmacy services across Europe. Longitudinal interpretation should take into account that the 2025 results included one additional country (33 vs 32 in 2020), and limited additions for newly emerging services. However, the core questionnaire structure, service definitions, response options, and implementation-level categories remained unchanged. In most cases (77%, n = 36), the community pharmacy services mapped in the study were reported to be available in a greater number of countries in 2025 compared with 2020, demonstrating a substantial broadening of service availability over the past five years. Notably, vaccination services increased from 10 to 19 countries, medication reconciliation from 7 to 15, and first-time dispensing interventions from 11 to 16 countries. Most community pharmacy services demonstrated either increase in geographical availability or increased levels of implementation intensity.

### Reimbursement trends

A general trend towards greater financial recognition of community pharmacy services was observed. The number of countries remunerating vaccination services doubled (5 to 10), while remuneration for screening at-risk individuals (2 to 6), and dose administration aids (5 to 8 countries) also increased. These results reflect an expanded policy commitment to implementing and sustaining remunerated community pharmacy services across Europe.

### Regulatory limitations

The survey recorded regulatory limitations in some services. These can take the form of extra regulations that have been enacted and in practice prevent pharmacists from applying their full potential in this service (e.g. generic substitution only allowed after consulting the prescribing doctor, or Home delivery of medicines only permitted to online pharmacies but forbidden for brick-and-mortar pharmacies); or legislation that is still not implemented (e.g. law changes required for services’ reimbursement). In other cases, legislation allows for partial provision of services or lack adequate implementation (e.g. services without implementing regulations and remuneration definition, despite the service formally existing in law). Limitations are particularly evident in pharmacist-delivered vaccination, where one third of countries offering this service report existing regulatory restrictions. In some countries legislation is still yet to be adopted to allow for pharmacists to vaccinate, despite vaccination being already allowed in community pharmacies by other healthcare professionals (e.g. Estonia and Sweden).

Across all services mapped, regulatory restrictions were reported in 22 out of 47 community pharmacy services, most commonly in clinical and substitution-related services (especially in dispensing services and individual case management services).

## Discussion

### Summary of key findings

This study features the wide range of community pharmacy services currently provided in the EU and beyond, that extend further from the traditional medicines dispensing role (Table 1). Many clinical and case-management services depend on legislative adaptations, with only some services being reimbursed by the governments. Particularly notable growth was observed in vaccination, medication reconciliation and first-time dispensing services.

The expansion observed since 2020 (as per Table 2) coincides with post-pandemic regulatory changes and broader health system reforms across Europe, which may have created favourable conditions for the development of new services. However, provided the observational design of this study, causal relationships cannot be definitively established. The COVID-19 pandemic period coincided with regulatory flexibility and increased public recognition of pharmacists as essential healthcare professionals in several countries. At the same time, increasing emphasis on primary care resilience, digital health strategies, and integrated service delivery, have created new opportunities for pharmacy-based interventions. These systemic shifts, combined with ongoing healthcare workforce shortages, have positioned community pharmacists as essential partners in improving access, continuity, and efficiency of care. Longitudinal evaluation of services delivery includes not only geographical availability increase, but also expansion in national-level implementation, moving from pilot to more structured interventions integrated into common practice.

These patterns have been summarised in Table 2, Figs. 1 and 2, which illustrate both geographical distribution and implementation intensity across the different pharmacy service categories.

### Vaccination

The increase in the number of countries offering pharmacy-based vaccination services is explained with the uptake of vaccination following the COVID-19 pandemic. In addition to this geographically availability increase, the number of countries reporting national-level service implementation increased too, which is a sign of not only wider adoption, but also greater integration in the routine pharmacy practice. Deslandes et al. found that in a study in Wales, community pharmacists were more likely than GPs to vaccinate those under 65 and “at risk”, a group in which flu vaccination rates are generally lower. People’s preference for using a pharmacy was mainly due to not needing to book an appointment, with high service acceptance and meeting service user needs. These results were shown without reducing the number of GP vaccinations [44]. Haems et al. examined community pharmacy vaccination across EU countries, demonstrating the transformative impact of COVID-19 on pharmacist-led vaccination policies. The authors identified four priority actions to advance community pharmacy vaccination policies: stronger pharmacists-policymakers collaboration, real-world evidence generation on pharmacist-led vaccination’ effectiveness, continuous professional development and training, and engagement with the public [45].

### Medication reconciliation

Expanding pharmacy-led medicines reconciliation services across Europe reflects an increased emphasis on patient safety, continuity of care, and transitions between healthcare settings [46]. Pharmacists’ increased involvement in reconciling medicines to avoid medication errors (interactions, duplications, dosing errors or omissions), especially when patients transition between levels of care, has been observed in several European countries [47]. European-wide studies and economic evaluations of related services (such as medication review) further support the clinical and cost-effectiveness potential of extending such services across community pharmacies [48]. The expansion observed in this study likely mirrors these developments and the growing collaboration between hospital and community pharmacists in post-discharge care [49].

### First-time dispensing intervention

The increase in countries implementing first-time dispensing interventions is consistent with the trend towards enhanced counselling and medicines optimisation at therapy initiation, defined as a central role for pharmacists. These interventions, where pharmacists provide structured advice to patients receiving a medicine for the first time, have shown positive outcomes in medication adherence, patient understanding, and therapy persistence [50, 51]. Evidence from national programmes has demonstrated improvements in adherence, as well as cost-effectiveness at the system level [52, 53]. The broader adoption of such interventions across Europe demonstrated in this study likely suggests policy shifts promoting pharmacists’ role in supporting safe and effective medicines use.

### Services reimbursement

Reimbursement remains a key enabler of pharmacy service implementation and sustainability across Europe. Some services are now publicly funded outside standard dispensing fees, particularly those related to prevention (vaccination, screening of at-risk patients) and therapy management goals (dose administration aids). Compared with 2020, public reimbursement increased for 49% (n = 23) of mapped services, with new funding areas emerging in therapy management (medicines reconciliation, deprescribing), prevention (pharmacy travel health), and health systems integration (scheduling visits/exams, delivery of reports, interprofessional activities).

Services reimbursement supports their implementation, service quality, and outreach [54]. Studies confirm that remunerated services achieve consistent delivery and greater patient uptake, and lack of payment remains a barrier to implementation [55]. Economic evaluations confirm their value, such as the UK New Medicines Service, which improved outcomes at reduced cost (€172 less per patient; + 0.05 quality-adjusted life year (QALYs)) [56]. Cost-effectiveness for Medicine Use Review and medication review services has been confirmed across Europe, with a positive perception by patients and pharmacists [52, 54, 57, 58].

National pilots further evidence cost-effectiveness. In Wales, the NHS-funded sore-throat test-and-treat service proved to be safe, effective, and cost-effective (24,000€ per QALY) [59, 60]. Similar conclusions from Denmark and Spain showcased measurable system-level benefits, especially in rural areas [52, 61]. An European-level review confirmed that community pharmacy services are cost-effective and contributed to the healthcare system’s economic benefit [40].

The presence of public/insurance reimbursement frameworks suggests increasing policy recognition of community pharmacy services. However, this study does not assess the adequacy or structure of remuneration mechanisms. In rural and underserved areas, pharmacies are often the most accessible healthcare point, offering significant value through improved coverage and accessibility [62, 63]. However, lower service volumes challenge financial viability. Regionally funded pilots have partially addressed this, but sustainable and differentiated contractual models are needed to maintain equity, quality, and long-term viability of service delivery [64].

The findings from this study suggest that expanding community pharmacy services availability alone may be insufficient, as regulatory alignment and adequate structural reimbursement frameworks are needed to transform implementation into sustained professional practice. Incorporating specific indicators on community pharmacy services’ integration and outcomes into health system performance assessments at international level (such as those conducted by OECD) could support benchmarking across countries, thus facilitating the identification of best practices. These comparative metrics would help leverage learning from practice, whilst reducing the need for each country to independently generate evidence, before implementing community pharmacy-based services [65].

### Barriers for the implementation of services

Regulatory limitations in national law continue to restrict the full implementation of community pharmacy services. Although pharmacists are recognised as healthcare professionals under European Legislation, in some countries they are not formally classified as healthcare providers (e.g. Estonia) or healthcare specialists (e.g. Lithuania), limiting regulatory progress and professional potential [66, 67].

Practical barriers, including heavy workloads, financial constraints, pharmacy organisation, limited staff, and high administrative burden, frequently hinder novel services integration into daily practice [68]. Kroenert and Bertsche identified time constraints, extensive documentation, inadequate remuneration, and communication challenges with patients and other professionals as major obstacles to implementing reimbursed community pharmacy services in Germany [69]. This has been confirmed in a recent review in OECD countries, highlighting time restraints, workload pressures, general practitioner resistance, limited consumer awareness, and remuneration as barriers for services implementation [70].

The regulatory and economic environment also shapes service quality and availability. Vogler et al. found pharmacies in deregulated markets may experience deterioration of services’ quality, whereas supportive frameworks promote expansion [71]. In an overview of systematic reviews, Fares et al. identify the need for policymakers to broaden pharmacists’ scope of practice, ensuring adequate reimbursement, to sustain high-quality and accessible services [72].

Further integration of community pharmacies within primary care is essential to optimise pharmacists skills, enhance patient outcomes, and promote safe and rational use of medicines [16, 27, 73]. Organisational and structural improvements, namely through standardised procedures, digitalisation, and improved data sharing, are needed to streamline workflows and enable pharmacies to function as integrated community health hubs amid growing demand and workforce pressures.

Expanding community pharmacy services also has implications for education and training, governance, and health systems integration. Pharmacy education and continuing professional development programmes will need to adapt in order to support clinical competencies and roles, interprofessional collaboration and digital skills [74]. With the expansion of pharmacists’ responsibilities, structured collaboration with healthcare professionals (in particular general practitioners) is crucial to ensure care coordination. Clear accountability and quality assurance mechanisms will be needed to monitor health outcomes and maintain patient safety. Finally, further integration of community pharmacists in primary care will require appropriate access to electronic health records, thus ensuring continuity and safe clinical decision-making.

Addressing these barriers requires a coordinated policy action, encompassing reform of the scope of practice, investment in workforce planning and capacity, and deeper integration of pharmacies in primary care.

### Strengths and weaknesses

This study’s main strengths lie in its high representativeness, being the first to collect evidence on community pharmacy services from all 27 EU countries and 6 Non-EU countries. It applied a robust data collection and validation process, using a previously validated tool to minimise bias to ensure consistency between the two sets of data collection. The methodological continuity supports direct comparisons between the 2020 and 2025 datasets. Limitations include reliance on reported information from a limited number of respondents per country, which may not fully reflect real practice and could introduce reporting bias. As the data relied on self-reporting from national professional organisations, the possibility of reporting bias cannot be fully excluded. However, responses were subject to internal verification and national validation to ensure both accuracy and consistency. Furthermore, in several cases multiple organisations and individuals from the same country contributed to the questionnaire response, allowing triangulation of information at national level. As professional pharmacists’ organisations (PGEU members) have extensive knowledge of service implementation (supported by both grey literature and peer-reviewed evidence) the data are considered exceptionally reliable and representative. Although service definitions were provided in the questionnaire (see Supplementary Material), the scope and implementation of these services may be subject to country variability. Cross-country comparisons reflect the presence and level of implementation of broadly defined service categories, instead of strict equivalence in their clinical content. This should be taken into account when interpreting differences between countries and over the time period, while still allowing for identification of wider structural and policy trends. Whilst methodological continuity supports consistent longitudinal comparison, minor contextual differences between 2020 and 2025 data may influence the interpretation of service change over time. The questionnaire did not collect data on payment mechanisms, fee levels, or perceived adequacy of remuneration; therefore, reimbursement analysis reflects solely the presence of public funding. Conclusions regarding the sustainability of remuneration models should be interpreted with caution.

### Further research

This study unveiled the differences in scope of practice for community pharmacy services existent in EU countries and beyond. Further research should prioritise three areas: firstly, to harmonise and standardise community pharmacy services; secondly, to assess long-term evaluation of clinical and cost-effectiveness outcomes, beyond pilot implementation of more complex interventions; and thirdly, to evaluate the structure and adequacy of community pharmacy services remuneration models in Europe, to ensure sustainable integration into primary care systems.

### Implications for policy and practice

This study highlights the need for the legal expansion of pharmacists’ scope of practice to support the continued development of community pharmacy services. Sustainable implementation requires appropriate remuneration mechanisms, and legislative frameworks for novel services should be piloted, implemented, and commissioned by governments. The capacity of community pharmacies to collect data and generate evidence should be further leveraged in the context of healthcare digitalisation, the European Health Data Space, and broader digital transformation. Strengthening pharmacists’ digital competencies, as proposed by Lee et al*.,* will be essential to this process [75]. Finally, community pharmacy networks can serve as strategic partners in public health preparedness and system-wide resilience planning.

## Conclusion

This study provides the first EU-wide evaluation of community pharmacy service delivery including all 27 EU Member States. While all countries reported implementation of community pharmacy services, there remains substantial variation in their number, scope, and implementation profile. The findings illustrate the broad range of services now provided in Europe, extending far beyond traditional dispensing roles. In several countries, pharmacists are now remunerated for specific services, marking a transition from a purely supply function to a service-based model of care. This evolution indicates increasing recognition of the expanding contribution of community pharmacies to health promotion, disease prevention, screening, and case management. Continued support from governments, particularly through appropriate remuneration frameworks, will be essential to sustain and further develop these services. Looking ahead, European community pharmacists are well positioned to contribute to patient safety, therapeutic optimisation, and the overall sustainability of the healthcare systems.

## Supplementary Information

Below is the link to the electronic supplementary material.

Supplementary file1 (DOCX 30 KB)

## Data Availability

The datasets generated during this study are available from the corresponding author on reasonable request.

## Declarations

### Data protection

All data were collected, processed, and stored in accordance with the European Union General Data Protection Regulation (GDPR). Questionnaire distribution and responses were managed through PGEU membership contact lists, and no personal or identifying information was retained.

### Artificial intelligence use

Artificial intelligence tools were used for minimal language editing during manuscript preparation. All scientific content, data analysis, interpretation, and conclusions were developed and verified by the authors.
