# Supplementary material for: Evolution of community pharmacy services in the European Union and beyond: a cross-country survey of 33 national pharmacy organisations
Source: Int J Clin Pharm. 2026 Apr 15;48(4):1559–72. doi: 10.1007/s11096-026-02137-9 (PMC13369040; doi:10.1007/s11096-026-02137-9)
Supplement: Supplementary file 1 — Supplementary file1 (DOCX 30 KB) [file 11096_2026_2137_MOESM1_ESM.docx]

# Supplementary Material

**Participating national pharmacy organisations in the PGEU Pharmacy Services Survey**

This supplementary material lists all national pharmacy organisations that contributed with data to the PGEU Pharmacy Services Survey, which collected information on the implementation, reimbursement, and regulation of pharmacy services across Europe. Data were gathered between June and December 2024 and validated with participating organisations in March 2025. The survey included all 27 EU Member States and several Non-EU countries. One non-PGEU member country, Lithuania, also provided a national response and is acknowledged for its voluntary participation.

Table S 1 - Participating national pharmacy organisations in the PGEU Pharmacy Services Survey

| PGEU member organisations | | |
| --- | --- | --- |
| Austria | Österreichische Apothekerkammer | |
| Austria | Österreichischer Apothekerverband | |
| Belgium | A.P.B - Association Pharmaceutique Belge/Algemene Pharmaceutische Bond | |
| Belgium | Orde der Apothekers – Ordre des Pharmaciens | |
| Bulgaria | Български фармацевтичен съюз | |
| Croatia | Hrvatska Ljekarnička Komora | |
| Cyprus | Παγκύπριoς Φαρμακευτικός Σύλλoγoς (ΠΦΣ) | |
| Czechia | Česká lékárnická komora | |
| Denmark | Danmarks Apotekerforening | |
| Estonia | Eesti Proviisorapteekide Liit | |
| Finland | Suomen Apteekkariliitto / Finlands Apotekareförbund | |
| France | Féderation des Syndicats Pharmaceutiques de France | |
| France | Ordre National des Pharmaciens - Conseil Central A | |
| France | USPO - Union des Syndicats de pharmaciens d'officine | |
| Germany | ABDA - Bundesvereinigung Deutscher Apothekerverbände | |
| Greece | Πανελλήνιος Φαρμακευτικός Σύλλογος | |
| Hungary | Magyar Gyógyszerész Kamara | |
| Ireland | Irish Pharmacy Union | |
| Italy | Federazione Ordini Farmacisti Italiani (FOFI) | |
| Italy | Federfarma | |
| Latvia | Aptieku īpašnieku asociācija | |
| Luxemburg | Syndicat des Pharmaciens Luxembourgeois a.s.b.l. | |
| Malta | Kamra ta`l-Ispiżjara ta` Malta | |
| Netherlands | KNMP - Koninklijke Nederlandse Maatschappij ter bevordering der Pharmacie | |
| North Macedonia | Фармацевтска комора на Македонија | |
| Norway | NAF – Apotekforeningen | |
| Poland | Naczelna Izba Aptekarska | |
| Portugal | Associação Nacional das Farmácias | |
| Portugal | Ordem dos Farmacêuticos | |
| Romania | Colegiul Farmacistilor din Romania | |
| Serbia | Farmaceutska komora Srbije | |
| Slovakia | Slovenská Lekárnická Komora | |
| Slovenia | Lekarniška Zbornica Slovenije | |
| Spain | Consejo General de Colegios Oficiales Farmacéuticos de España | |
| Sweden | Sveriges Apoteksförening | |
| Switzerland | Société Suisse des Pharmaciens pharmaSuisse | |
| Turkey | Türk Eczacıları Birliği | |
| United Kingdom | National Pharmacy Association | |
| United Kingdom | Pharmaceutical Society of Northern Ireland | |
|  | | |
| Non-PGEU member organisations | | |
| Lithuania | | Lietuvos Vaistinių Asociacija |

**Pharmacy Services definition**

The following definitions of pharmacy services were used in the survey, and are listed in alphabetical order:

**Access to Patient Medical Records or Summaries**: Access by community pharmacists to electronic patient medical records (electronic health records / EHR), with patients’ consent, usually with read and/or write privileges.

**Administering injectable medicines**: E.g. antibiotics or adrenaline when required.

**Chronic disease management**: A multi-component pharmacy intervention aiming to control symptoms and slow or stop chronic disease progression. This can include the detection of early signs or changes in a patient's condition, medication monitoring, medication review, adherence support and disease self-care support.

**Common Ailment Management Schemes**: Providing medicines and advice to patients with common health conditions so they do not need to visit a doctor to receive them. It can include the option to prescribe over-the-counter (OTC) and/or prescription-only medicines by the pharmacist.

**Data collection on the effectiveness of new medicines**: Pharmacists involvement in Health Technology Assessment.

**Deprescribing**: Identifying and withdrawing medication when the risks of the medicine outweigh its clinical benefits (e.g., benzodiazepines, antidepressants)

**Directly observed treatment/supervised consumption of medicines**: E.g. Methadone, buprenorphine, antibiotics, medicines for the treatment of tuberculosis, HIV etc., others

**Dose Administration Aid**: Dose Administration Aids (DAA) are provided with the aim of assisting people with the management and timing of their medicines and can include a repackaging scheme.

**Drug dose titration**: Drug titration is the process of adjusting the dose of a medication for the maximum benefit without adverse effects.

**Emergency Contraception**: The possibility for pharmacists to dispense, prescribe, or supply emergency contraception to the population.

**Emergency supply of prescription-only medicines without prescription**: Supply and/or administration of a prescription medication upon request from a patient previously prescribed it, in an emergency, without a prescription (e.g., adrenaline or salbutamol, or other life-saving medicines).

**First time dispensing intervention**: A structured, private consultation between a pharmacist and a patient starting a new medication focusing on supporting adherence in the first months of treatment.

**Galenic formulation / Compounding**: Preparation of either magistral or officinal formulas involving compounding techniques.

**Generic substitution**: The practice of exchanging at pharmacy level one medicine instead of another with the same active substance, strength and pharmaceutical from another manufacturer, without consulting the prescriber.

**Handling and disposal of expired or unwanted medicines**: Disposal of Medicines Waste includes safe disposal of expired or un-used medicines in pharmacy.

**Health education**: Promoting public health through campaigns, events or counselling.

**High-Cost Therapy Dispensing and Management**: Dispensing high-value medicines (e.g., biological or biosimilar medicines) to patients in the community pharmacy (e.g., Oncology, HIV, Rheumatoid Arthritis, Multiple Sclerosis).

**Home delivery**: the home delivery service allows for the delivery of medicines when no other person is able to collect the item from the pharmacy and deliver it to the patient, ensuring continuous supply of medicines.

**Home or Nursing Home Medication Review**: Medicines review performed at patients’ homes or in nursing homes by a community pharmacist.

**In pharmacy point of patient access to health services**: Possibility for patients to access other health services in the pharmacy premises / through pharmacies.

**Information to patients on conditions / treatments**: Advanced counselling that pharmacists provide to patients on specific conditions and treatments.

**Instruction on the use of therapeutic, self-monitoring device or medical aid**: Teaching patients how to use certain devices or software in order to achieve the best health results. Some devices have demonstration devices to be present at the pharmacy such as insulin pens or inhalers.

**Integrated care pathways/protocols in place with primary care**: Integrated care pathways are complex multidisciplinary plans of care that stipulates the necessary steps for managing patients with particular clinical conditions.

**Medication Reconciliation**: Medication reconciliation is the process of comparing a patient's medication orders to all of the medications that the patient has been taking. This reconciliation is done to avoid medication errors such as omissions, duplications, dosing errors, or interactions with other medicines.

**Medication Review**: A structured evaluation of patients’ medicines with the aim of optimising medicines’ use and improving health outcomes. This entails detecting drug related problems and recommending interventions.

**Mobile applications**: Development of mobile applications (apps) aiming to aid therapeutic management, pharmacy’s location and general information, and access to services.

**Needle/syringe exchange**: Service where intravenous drug users can obtain clean injecting equipment, support, and information. It is usually complemented by support for self-care and referral to another health or social care professional if needed.

**Night services**: Night Services are on-call or extended opening services that increase the accessibility to community pharmacies.

**Off-site multi-professional activities**: These activities generally involve meetings (outside pharmacy premises) with other healthcare professionals.

**Pharmacist-delivered vaccination**: Administration of vaccines by the community pharmacist, usually inside the pharmacy premises.

**Pharmacovigilance for medicines under additional monitoring**: Medicines under additional monitoring have a black inverted triangle displayed in their package leaflet (▼). Additional monitoring aims to enhance reporting of suspected adverse drug reactions for medicines for which the clinical evidence base is less well developed. The main goals are to collect information as early as possible to further inform the safe and effective use of these medicines and their benefit-risk profile when used in everyday practice. In some countries the scope is broader and this service might apply to all medicines.

**Pharmacy travel health**: Pre-travel consultation conducted by pharmacists that can include the dispensing and/or administration of prescription medicines (e.g. vaccines) for travel purposes.

**Predictive medicine**: The identification of patients at risk of developing a disease with the aim of pinpointing targets for prevention or early intervention of the disease.

**Referral to other healthcare providers**: If certain referral criteria are present, pharmacists can structurally refer patients to other healthcare professionals (e.g. specialists medical doctors), accompanied by communication to the referral healthcare professional.

**Refusal to dispense due to safety reasons**: The process through which pharmacists can opt not to dispense a medicine (usually a prescription-only medicine) based on their clinical (or other) assessment.

**Repeat dispensing**: System/service whereby repeat issues of prescriptions (e.g., for medications for chronic diseases) are managed (held) by the pharmacy and dispensed when required/due to the patient without needing to request a valid prescription from the doctor (i.e., the pharmacy holds future issues of the prescription in the pharmacy which are pre-authorised by the prescriber for a set duration (e.g., 3, 6 or 12 months).

**Scheduling visits/exams, delivery of reports**: Patients take advantage of community pharmacies as facilities providing healthcare, being able to schedule consultations with other healthcare structures, and access health reports transferred from other levels of care.

**Screening individuals at-risk not on medication**: Performing a rapid diagnostic test/screening service by the pharmacist and/or providing people the opportunity to perform the rapid diagnostic test themselves inside the pharmacy, e.g., for diabetes, hypertension, lipid disorder, HIV, Hep B, Hep C, Strep-A, colon cancer. This service may apply to both screening for a disease or early detection.

**Shared Electronic Patient Medication Records**: Access by community pharmacists to electronic medication records, with patients’ consent, usually with read and/or write privileges.

**Smoking cessation**: Performing counselling to help people give up smoking.

**Supply of self-test kits to the public**: Supply/selling of point-of-care tests or kits to the general public for disease screening purposes.

**Tele/online consultations with other healthcare providers**: Usually this service involves pharmacists’ consultation with other healthcare professionals through electronic means, in the scope of a patients’ therapeutic discussion.

**Teleconsultations by pharmacists**: The possibility for pharmacists to consult and provide patients/public with advice by phone or online.

**Therapeutic adherence support**: Structured service/program to identify possible issues related to therapy adherence and provide solutions and motivation.

**Therapeutic** **Substitution**: The practice of exchanging at pharmacy level one medicine instead of another with a different active substance and with the same therapeutic intent, in consultation with the prescriber and patient or in accordance with national/local protocols.

**Urgent supply of prescription-only medicines without prescription**: Supply of a prescription medication upon request from a patient who had been previously prescribed with it, in case of urgency, without a prescription (e.g., patient on holiday or ran out of medicine and cannot obtain a prescription).

**Weight management**: Service where patients can take advantage of the easy access and informal environment of community pharmacies to follow weight management programmes.
